# Aging Aggravates Periodontal Inflammatory Responses and Alveolar Bone Resorption by *Porphyromonas gingivalis* Infection

**DOI:** 10.3390/cimb45080416

**Published:** 2023-08-08

**Authors:** Yuri Song, Jin Chung

**Affiliations:** 1 Department of Oral Microbiology, School of Dentistry, Pusan National University, Yangsan 50612, Republic of Korea; luckcute@naver.com; 2 Oral Genomics Research Center, Pusan National University, Yangsan 50612, Republic of Korea

**Keywords:** *Porphyromonas gingivalis*, inflammasome, cytokines, periodontitis, aging, macrophages

## Abstract

Periodontitis is a chronic inflammatory disease driven by periodontal pathogens such as *Porphyromonas gingivalis (P. gingivalis),* and its prevalence increases with age. However, little is known about the effect of immunosenescence on inflammatory response to *P. gingivalis* infection. In the present study, 16S rDNA sequencing analysis showed the relative abundance of *P. gingivalis* was significantly higher in periodontitis patients than healthy group, but there was no difference between the young (20 to 40 years old) and old (65 to 86 years old) periodontitis groups. Furthermore, the cytotoxic effect of P. gingivalis was greater on old THP-1 macrophages and on bone mar-row-derived cells (BMDMs) from old mice, and levels of interleukin (IL)-1β, tumor necrosis factor (TNF)-α, and IL-12 were higher in old than in young THP-1 macrophages. Furthermore, the activations of inflammasome components for IL-1β production by *P. gingivalis* infection were greater in old THP-1 macrophages. Finally, bone loss was significantly greater in *P. gingivalis*-infected aged mice than in young mice. These findings indicate that aging aggravates *P. gingivalis*-induced inflammatory cytokine secretion and inflammasome activation. The study enhances understanding of the relationship between periodontal immunosenescence and inflammatory response in the elderly.

## 1. Introduction

Periodontitis is induced by microbial biofilms and causes host-mediated inflammation, leading to tissue damage and tooth loss [1]. Balance between resident microbiota and host immune response maintains a state of homeostasis, while disruption of this balance leads to inflammation and disease progression [2]. As lesions develop, alveolar bone height gradually decreases, tooth foundations weaken, tooth roots are exposed, and it left untreated, tooth loss occurs [3]. In particular, periodontitis is more prevalent and severer in older adults [4]. Futhermores, the oral microbiome changes with aging, and older adults have higher levels of some Gram-negative bacilli, such as *Porphyromonas endodontalis*, *Alloprevotella tannerae*, and *Filifactor alocis* in the saliva [5]. Recently, it was reported that the oral colonization by *P. gingivalis* increases with age [6]. *P. gingivalis*, an anaerobic Gram-negative rod, is the major pathogen for periodontitis [7]. *P. gingivalis* expresses several virulence factors, including lipopolysaccharides (LPSs), fimbriae, and gingipain that may support its survival, regulate its communication with other species in biofilms, and modulate the inflammatory response of the colonized host tissue [8].

Macrophages in the periodontal tissue play a central role in front-line immune response to invading pathogens during the initiation and progression of periodontal disease [9]. Macrophages eliminate pathogens by phagocytosis or secrete cytokines that induce inflammatory response [10], and have antigen-presenting functions that signal acquired immunity [11]. During infection resolution, macrophages also act to downregulate inflammatory cytokines and promote tissue repair, but if inflammatory cytokines production is excessive and results in uncontrolled accumulation, irreversible loss of attachment and disruption of the alveolar bone occurs [12]. 

Aging is associated with alterations in the homeostasis of cellular maintenance and repair processes [13]. The age-related impairment of immune response is referred to as immunosenescence and has been reported in monocytes, macrophages, dendritic cells, and T cells [14]. Immunosenescence leads to a susceptibility to microbial infections, and causes low-grade, chronic, systemic inflammation referred to as ‘inflammaging’ [15]. Moreover, it has been reported that human monocytes from older individuals exhibit higher levels of oxidative stress and secrete more inflammatory cytokines (e.g., TNF-α and IL-1β) [16]. Previously, we reported that *P. gingivalis* activates the NLR family pyrin domain-containing 3 (NLRP3)/ absent in melanoma 2 (AIM2) inflammasome complex, and thus, induces IL-1β secretion [17]. IL-1β is produced in cells as cytosolic pro-forms and secreted in tis active forms after inflammasomes cleavage [18]. An inflammasome complex is composed of nucleotide-binding domain leucine-rich repeat-containing (NLR) or AIM2 family receptors, apoptosis-associated speck-like protein (ASC), and procaspase-1 [19]. The severity of periodontitis is known to be associated with aging, but the effects of aging on periodontal immune response to pathogens have not been fully elucidated. 

The objectives of the present study were to investigate the effects of aging on inflammation in *P. gingivalis*-infected macrophages, and to determine whether aging affects alveolar bone loss in a *P. gingivalis*-induced periodontitis mice model.

## 2. Materials and Methods

### 2.1. Clinical Sampling

16S rRNA datasets and the relative abundances of *P. gingivalis* in healthy subjects and periodontitis patients were obtained from a previous study (PMID: 32443919). Clinical samples were obtained from healthy subjects and patients that visited the Periodontology Department of Pusan National Dental School. All subjects were provided written informed consent, and their rights are protected by the Institutional Review Board of Pusan National University (IRB No. PNUDH-2017-023). Periodontitis was diagnosed as per the guidelines set out by the 2017 World Workshop on the Classification of Periodontal and Peri-implant Diseases and Conditions [20] and used in previous studies [21]. Briefly, full-mouth clinical examinations, including probing depth (PD), clinical attachment level (CAL), gingival index (GI), and plaque index (PI) assessments, were conducted by one practitioner. Patients with periodontitis had moderate-to-severe periodontal disease, including PD > 3 mm, clinical attachment loss > 3 mm, and radiographic evidence of distinct bone loss. The healthy control (H) group consisted of individuals with clinically healthy gingival tissues (low scores of bleeding on probing in <10% of the sites and no sites with PD > 3 mm or CAL).

For sampling plaque, all participants were requested to refrain from food for 2 h and oral hygiene (brushing or flossing teeth) for 2 h before sampling. Buccal swab samples were obtained from the mucosa of both cheeks. Supragingival plaque samples were collected from the mesiobuccal surface of upper or lower 1st molars. Samples were collected after isolating the selected sampling site with cotton rolls and air drying gently. Each samples was collected with a sterile microbrush and placed in a separate, sterile 1.5 mL microcentrifuge tube. Plaque samples were stored at −80 °C until required.

The data of 114 subjects, that is, 55 healthy subjects (20–40 years) and 59 patients with periodontitis (20–40 years (N = 29) and 65–86 years (N = 30)), were analyzed. Wilcoxon’s rank sum test was used to determine the significances of intergroup differences. The normalities of the distributions of these four clinical parameters were determined using Shapiro-Wilk normality tests. However, all were non-normally distributed, and thus Kruskal–Wallis tests used to compare parameters in the three groups.

### 2.2. Mice 

SMP30 knockout (KO) mice, provided by Dr. Hae-Young Chung, College of Pharmacy of Pusan National University, were used to study the effects of aging on *P. gingivalis*-induced periodontal inflammatory response [22]. SMP30 KO mice (6 weeks) were divided into two groups; (1) young mice were administered vitamin C in tap water at 1.5 g/L for 8 weeks and (2) old mice were provided tap water without vitamin C for 8 weeks. All mice were maintained at 23 ± 2 °C with a relative humidity of 60 ± 5% and a 12 h light–dark cycle. All animal studies were approved by the Institutional Animal Care Committee of Pusan National University. The study adhered to all guidelines for animal experiments issued by Pusan National University (Approval Number PNU-2022–0108).

At 14 weeks, bone-marrow-derived cells (BMDMs) were isolated from femurs and tibias and differentiated to macrophages by culture in the conditioned medium of L929 cells, which contained a macrophage colony-stimulating factor (M-SCF) solution for 4 days. Non-adherent cells then were removed, and the adherent cells were used in subsequent experiments.

The *P. gingivalis*-induced periodontitis mice model was generated by inoculating mice with *P. gingivalis* (strain 381) at 14 weeks. All mice were administered sulfamethoxazole-trimethoprim ad libitum in drinking water to remove the resident oral flora. Mice were rested for three days before inoculations and then were orally administered 10^9^ colony-forming units of *P. gingivalis* suspended in 100 μL of 2% carboxymethyl cellulose in sterile phosphate-buffered saline (PBS; Sigma Aldrich, St. Louis, MO, USA) 3 times weekly for 2 weeks. Mice were euthanized at 18 weeks, and the alveolar bone resorption were measured. 

### 2.3. Cell Culture 

THP-1 monocytes were cultured in RPMI (Thermo Fisher Scientific, Waltham, MA, USA) supplemented with 10% fetal bovine serum and differentiated into macrophages by treating them with 50 nM phorbol 12-myristate 13-acetate (PMA; Sigma Aldrich) overnight. 

To generate the old cells, THP-1 macrophages were aged for 6 days in medium containing 100 μM of bromodeoxyuridine (BrdU; Sigma Aldrich), which was exchanged every 2 days. 

### 2.4. Bacterial Culture 

*P. gingivalis* (strain 381) was purchased from ATCC (Manassas, VA, USA) and cultured anaerobically in Gifu anaerobic medium (GAM; Nissui, Tokyo, Japan) containing 5 mg/mL hemin and 0.5 mg/mL 3-phytyl-menadione (vitamin K) in an 80% nitrogen, 10% hydrogen, and 10% CO_2_ anaerobic system (Anoxomat; Advanced instruments, Norwood, MA, USA). *P. gingivalis* was collected by centrifugation at 5000 rpm for 5 min and resuspended in PBS to infect cells at the indicated multiplicity of infection (MOI) for 24 h. 

### 2.5. Cell Death Assay

Cell death was measured using a lactate dehydrogenase (LDH) cytotoxicity assay kit (Promega, Madison, WI, USA). Briefly, cell supernatants were mixed with lactate dehydrogenase (LDH) buffer for 30 min, and absorbance were measured using a spectrophotometer at 492 nm.

### 2.6. Cytokine Assay 

The levels of IL-1β, IL-8, and IL-12 were measured using an ELISA kit (BioLegend, San Diego, CA, USA), following the manufacturer’s instructions. Cytokine levels were determined by measuring absorptions at 450/570 nm using a spectrophotometer (Tecan, Männedorf, Switzerland). 

### 2.7. Western Blot Analysis

Cells were harvested and lysed in RIPA buffer (Cell Signaling Technology, Danvers, MA, USA) containing protease inhibitor cocktail (Sigma-Aldrich). Protein samples were separated using 10–15% SDS-PAGE and then transferred to membranes (MilliporeSigma, Burlington, MA, USA), which were then probed with primary and secondary antibodies. Membranes were developed using a chemiluminescence solution (GE Healthcare, Chicago, IL, USA) in a LAS-4000 Lumino-imaging unit (Fujifilm, Tokyo, Japan). Immunoblot band intensities were quantified using NIH ImageJ 1.54f software (Fujifilm), and the results are presented as intensity ratios versus β-actin. The antibodies used were as follows: anti-β-actin (Santa Cruz Biotechnology, Dallas, TX, USA), anti-human AIM2 (Cell Signaling Technology), anti-human ASC (Cell Signaling Technology), pro-Caspase-1 (Cell Signaling Technology), anti-human IL-1β (R&D Systems, Minneapolis, MN, USA), and NLRP3 (NOVUS biologicals, Centennial, CO, USA) 

### 2.8. Confocal Laser Scanning Microscopy for ASC Speck Observation

THP-1/ASC-GFP cells were seeded in 8-well chambers (Sigma Aldrich) and challenged with *P. gingivalis*. The formation of ASC speck was observed by confocal laser scanning microscopy (LSM 700; Carl Zeiss, Oberkochen, Germany), and quantified by expressing the number of cells containing ASC as percentages of the total cells.

### 2.9. Micro-CT Scanning and Assessment of Alveolar Bone

Mice mandibular bone were prepared to measure alveolar bone resorption. Three-dimensional images were scanned by micro-CT (InspeXio SMX-90CT; Shimadzu Science, Tokyo, Japan) in slices at 90 kV, 110 μA, and 0.5 mm aluminum attenuation filter. Scans were reconstructed to generate three-dimensional models. Regions of interest (ROI) were cuboidal bone bodies, including roots. ROI region included the most mesial to the most distal aspects of the lower three molar roots. Three-dimensional images were prepared in a standardized anatomical reference position using TRI/3D Bone (Ratoc, Tokyo, Janpan). AmountS of lost alveolar bone were obtained by summing areas from the cemento-enamel junction (CEJ) to the alveolar bone crest (ABC) of each teeth using ImageJ. A single-blinded examiner performed all measurements. 

### 2.10. Statistical Analysis

Variable normalities were checked using the Shapiro–Wilk test. Normally distributed variables were ana-lyzed using the Student’s *t*-test, and non-normally distributed variables using the Mann-Whitney test. One-way analysis of variance (ANOVA) with Dunnett’s post-hoc test was used to compare multiple groups. The analysis was conducted using GraphPad Prism 9.5 software (GraphPad, San Diego, CA, USA), and re-sults are presented as means ± SDs. Statistical significance was accepted for *p* values < 0.05.

## 3. Results

### 3.1. P. gingivalis Abundances Were Elevated in Young and Old Periodontitis Patients

Clinical samples were obtained for healthy subjects (control) and young (20–40 years old) and old (65–86 years old) periodontitis patients. When the four clinical parameters were compared, all showed significant intergroup differences (Table 1). Clinical indices (mean PD, CAL, GI, and PI) were all significantly higher in the periodontitis groups than in the the control group. In particular, mean CAL was increased from 2.34 mm in the control group to 4.46 and 3.65 mm in the young and old periodontitis groups, respectively. 

**Figure 1:**
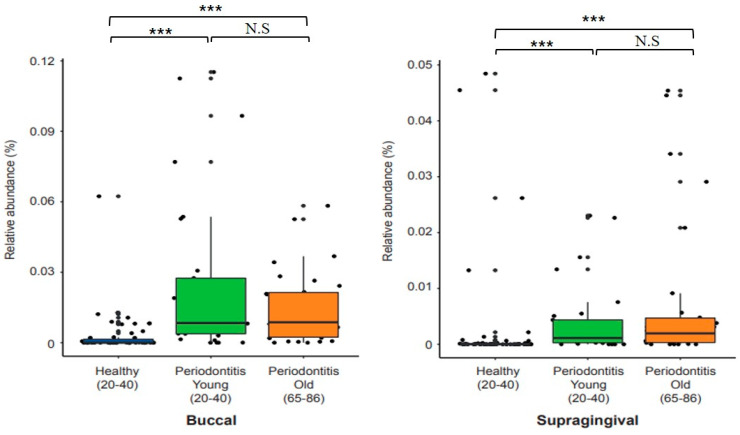
The relative abundance of *P. gingivalis* in the clinical samples by age.The relative abundance of *P. gingivalis* in the plaque samples of buccal mucosa and supragingival sites was analyzed using the 16S rRNA sequencing data (PMID: 32443919). Violin plots were drawn for healthy subjects and young and old periodontitis patients. Left panel: buccal mucosa sites; right panel: supragingival sites. Wilcoxon rank sum tests were used to determine the significance of the differences. *** *p* < 0.001 control of Healthy versus Periodontitis patients; N.S = Not Significance.

Next, 16s rDNA sequencing was used to check the effect of aging on the relative abundance of *P. gingivalis* in dental plaque in the three groups. Relative abundances of *P. gingivalis* were significantly greater in buccal and supragingival plaque samples of both the young and old periodontitis groups than in the control group (Figure 1), but no significant difference was observed between young and old patients. These results con-firmed that dental plaque from young and old periodontitis is colonized to a greater extent by *P. gingivalis* than dental plaque from healthy subjects.

### 3.2. Aging Enhanced the Cell Death Induced by P. gingivalis Infection

**Figure 2:**
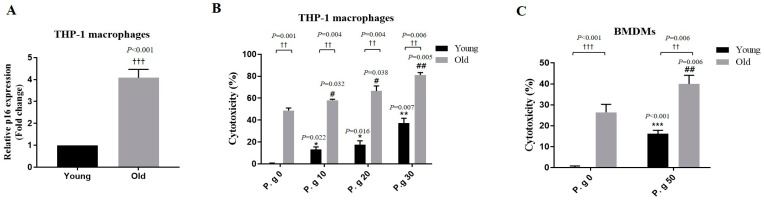
Effect of age on the cytotoxic effect of *P. gingivalis* infection. (**A**) THP-1 macrophages were treated with a medium containing BrdU for 6 days, and p16 mRNA levels were analyzed by a real-time PCR. Relative mRNA expression levels were normalized to those of GAPDH. (**B**) Young and old THP-1 macrophages and (**C**) young and old BMDMs were infected with *P. gingivalis* for 24 h, and cell viabilities were measured using an LDH assays. Results are presented as the means ± SDs (N = 6). Results are representative of three individual experiments. * *p* < 0.05, ** *p* < 0.01, *** *p* < 0.001 control of young cells versus *P. gingivalis*-infected young cells; ^#^ *p* < 0.05, ^##^ *p* < 0.01 control of old cells versus *P. gingivalis*-infected old cells; ^††^ *p* < 0.01, ^†††^ *p* < 0.001 young cells versus old cells.

The cytotoxic effect of *P. gingivalis* on young and old cells was examined uging an LDH assay to confrim the effect of aging on P. gingivalis–infected cells. We first checked the mRNA level of p16, a known biomarker of aging in young and old THP-1 macrophages. As was expected, the expression of p16 increased in old THP-1 macrophages (Figure 2A). Furthmore, the cytotoxic effect of P. gingivalis infection was greater on old than on young THP-1 macrophages (Figure 2B). Similarly, the cell death of old THP-1 BMDMs infected with *P. gingivalis* increased compared to that of young THP-1 BMDMs (Figure 2C). These results indicate that aging enhances *P. gingivalis*-induced cytotoxicity.

### 3.3. Aging Enhanced the Expressions of Inflammatory Cytokines Induced by P. gingivalis Infection

**Figure 3:**
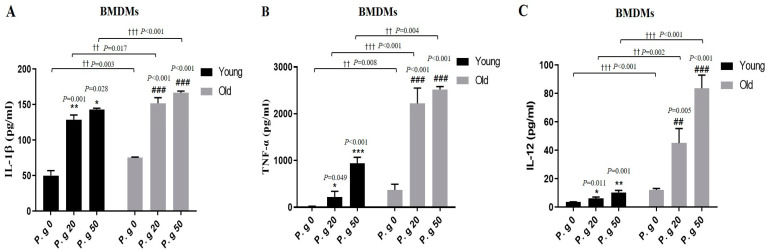
Effect of age on *P. gingivalis* infection-induced cytokine production. (**A**–**C**) Young and old BMDMs were infected with *P. gingivalis* (MOI 20 and 50) for 24 h, and culture supernatants were then assayed for IL-1β, TNF-α, and IL-12 by ELISA. Results represent means ± SDs (N = 6). Results are representative of three individual experiments. * *p* < 0.05, ** *p* < 0.01, *** *p* < 0.001 control of young cells versus *P. gingivalis*-infected young cells; ^##^ *p* < 0.01, ^###^ *p* < 0.001 control of old cells versus *P. gingivalis*-infected old cells; ^††^ *p* < 0.01, ^†††^ *p* < 0.001 young cells versus old cells.

We investigated the impact of aging on inflammatory cytokine production induced by *P. gingivalis* in BMDMs by performing ELISA on young and old cells. Levels of the pro-inflammatory cytokines, TNF-α and IL-1α, in young BMDMs were increased by *P. gingivalis* infection in an MOI-dependent manner and were significantly more in old BMDMs than in young BMDMs (Figure 3A,B). IL-12 levels were increased slightly more by *P. gingivalis* infection in old BMDMs than in young BMDMs (Figure 3C). These results indicate that aging enhances *P. gingivalis*-induced increases in the expression of inflammatory cytokines.

### 3.4. Aging Enhanced the Activations of NLRP3 and AIM2 Inflammasome Induced by P. gingivalis Infection

**Figure 4:**
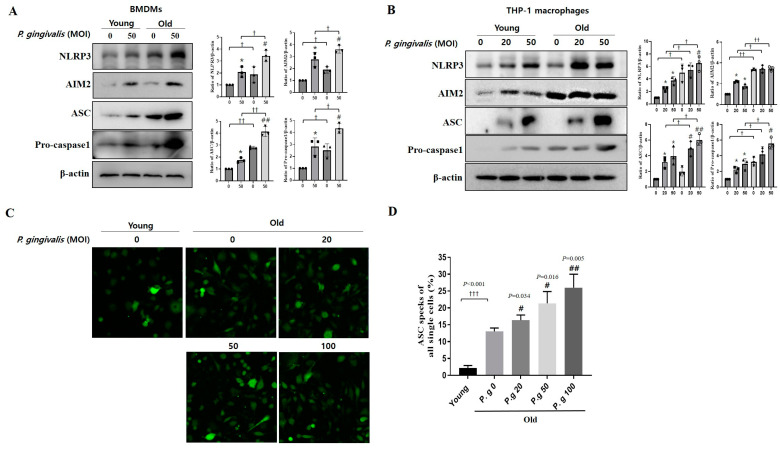
Effect of age on the activation of NLRP3/AIM2 inflammasome induced by *P. gingivalis* infection. (**A**) Young and old BMDMs were infected with *P. gingivalis* (MOI 50) for 24 h. (**B**) Young and old THP-1 macrophages were infected with *P. gingivalis* (MOI 20 and 50) for 24 h. The expressions of AIM2, ASC, and pro-caspase1 were assessed by immunoblotting. Densitometry of the band intensity was performed. (**C**,**D**) Young and old ASC-GFP/THP-1 macrophages infected with *P. gingivalis*. ASC specks were observed by fluorescence confocal microscopy. (**C**) Confocal images (×200 magnification). (**D**) Plots of the percentages of cells containing ASC specks. Results represent means ± SDs (N = 6). Results are representative of three individual experiments. * *p* < 0.05 control of young cells versus *P. gingivalis*-infected young cells; ^#^ *p* < 0.05, ^##^ *p* < 0.01 control of old cells versus *P. gingivalis*-infected old cells; ^†^ *p* < 0.05, ^††^ *p* < 0.01, ^†††^ *p* < 0.001 young cells versus old cells.

To investigate the effect of aging on the mechanism of increased IL-1β production induced by *P. gingivalis* infection, we examined the activation of inflammasome using a Western blot assay. The expression of NLRP3, AIM2, ASC, and pro-caspase-1 in old BMDMs infected with *P. gingivalis* was higher than that of young BMDMs (Figure 4A). Similarly, the expressions of NLRP3, AIM2, ASC, and pro-caspase-1 in old THP-1 macrophages infected with *P. gingivalis* was higher than those in young THP-1 macrophages (Figure 4B). 

Next, to evaluate the effect of aging on the formation of ASC specks, ASC speck formation was observed in ASC-GFP/THP-1 macrophages using a confocal microscope. because ASC forms a single supramolecular assembly within mac-rophages subjected to inflammatory stimuli. ASC specks were observed as bright spots in the cytoplasm of infected THP-1 macrophages. In non-infected cells, ASC speck formation was 5 times greater in old cells than in young cells. Furthermore, *P. gingivalis* infection increased the number of ASC specks MOI-dependently in old cells (Figure 4C,D). These results indicated that aging enhanced the activations of NLRP3 and AIM2 inflammasome induced by *P. gingivalis* infection. 

### 3.5. Aging Aggravated Alveolar Bone Resorption in P. gingivalis-Infected Periodontitis Mice

**Figure 5:**
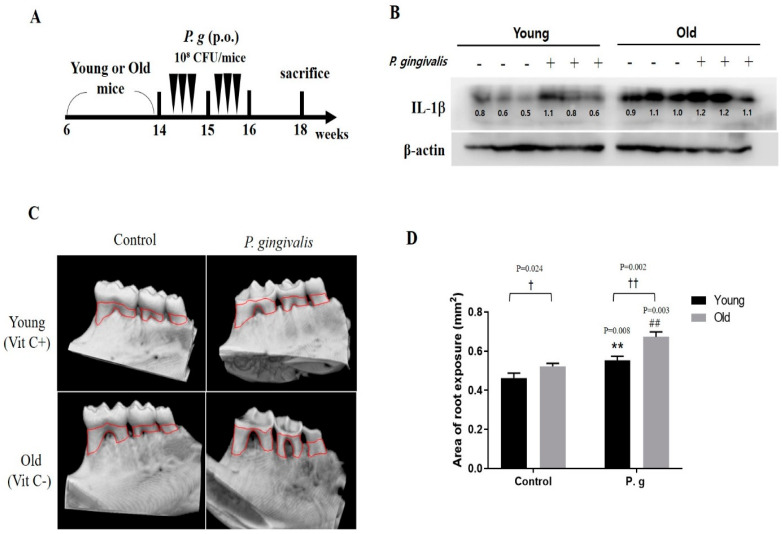
The alveolar bone resorption in *P. gingivalis*-infected mice by age. (**A**) Experimental design for periodontitis mice models (N = 6–7). (**B**) The protein expression of IL-1β was determined by Western blot. (**C**) Mandibular alveolar bone was scanned by micro-CT. (**D**) Total bone losses. Bar graphs represent means values ± SDs. ** *p* < 0.01 control of young mice versus *P. gingivalis*-infected young mice; ^##^ *p* < 0.01 control of old mice versus *P. gingivalis*-infected old mice; ^†^ *p* < 0.05, ^††^ *p* < 0.01 young mice versus old mice.

A periodontitis model was developed by inoculating *P. gingivalis* into young (vitamin C+) and old (vitamin C‒) mice (Figure 5A) to investigate the effects of aging. The protein expression of IL-1β was greater in the cervical lymph nodes of old mice than in those of young mice, but was not affected by *P. gingivalis* infection (Figure 5B). Micro-CT showed more severe bone resorption in the control of the old mice than that of young mice (Figure 5C,D). Of note, Two-way ANOVA revealed a significant association between age and *P. gingivalis*-induced periodontitis despite a significant difference between alveolar bone resorption in non-infected young and old mice at baseline.

## 4. Discussion

Periodontitis is a chronic inflammatory disease caused by microbial infection that ultimately destroys periodontal tissues [1]. Aging is characterized by a progressive loss of physiological integrity and includes physiological, structural, and functional changes [23], and age-related changes in the periodontal tissue occur in older adults, but do not lead to severe periodontitis [24]. Immunosenescence is an immune dysfunction that results in increased susceptibility to microbial infections and is closely related to various diseases, such as autoimmune diseases, malignant tumors, Alzheimer’s disease, and periodontitis [25,26,27]. Macrophages play an important role in immune response by regulating inflammatory cytokine levels, and senescent macrophages exhibit less phagocytosis, and elevated levels of senescent-associated markers and inflammatory cytokines [28]. The goal of this study was to investigate the impact of aging on immune responses to *P. gingivalis* infection in macrophages and mice. The effect of aging on *P. gingivalis* infection was investigated by comparing inflammatory cytokines production, inflammasome formation, and bone resorption in old versus young cells and mice.

Initially, we analyzed clinical parameters and amounts of *P. gingivalis* in clinical periodontitis patients. CAL values were higher for periodontitis patients than the healthy controls (Table 1). As for the relative abundance of *P. gingivalis*, as was expected, it was significantly greater in the periodontitis patients. However, no significant difference was observed between young and old periodontitis patients (Figure 1), which contrasts with previous results. For example, the abundance of *P. gingivalis* was reported to increase with age (14–70 years) in severe and refractory periodontitis [29], and the abundances of *A. actinomycetemcomitans* and *P. gingivalis* in subgingival plaque also reported to increase with age [30]. This discrepancy may have benen due to the use of different analytical methods or the sampling locations. Nonethelss, *P. gingivalis* was significantly more abundant in the older adults than in the healthy group.

To determine whether severe periodontitis in older adults is associated with immunosenescence, we examined the cytotoxic effects of *P. gingivalis* on young and old macrophages. As was expected, *P. gingivalis*-induced THP-1, and BMDM cell death was more severe in old macrophages, indicating that inflammation and aging enhanced *P. gingivalis*-induced cell death (Figure 2). Macrophage death by apoptosis, pyroptosis, or necroptosis is tightly regulated [31], but when uncontrolled, age-induced macrophage death causes tissue damage and accelerates the progression of inflammatory diseases such as periodontitis [31]. Our results showed that *P. gingivalis* is more cytotoxic to old cells.

Next, we examined the effects of *P. gingivalis* on inflammatory cytokine expressions in young and old BMDMs (Figure 3). Unfortunately, treatment with BrdU plus *P. gingivalis* reduced THP-1 macrophage sur-vival to ~20% (Figure 2B), which prevented our measuring cytokine levels. On the other hand, the cytokine levels in old non-infected BMDMs were higher than in their young counterparts, and levels of all cytokines in old infected BMDMs were higher than in young infected BMDMs. Pro-inflammatory cytokine secretion is one of the most important macrophage responses to periodontal pathogens [32], but cytokine overexpres-sion activates osteoclasts and causes alveolar bone absorption [33]. It was also reported that levels of peripheral cytokines, such as IL-1β, TNF-α, IL-6, and C reactive protein, were significantly higher in elderly with Alzheimer’s disease [34]. In the present study, IL-12 levels were slightly higher in young infected BMDMs than in non-infected controls but considerably higher in old BMDMs (Figure 3C). IL-12 promotes effective adaptive immune response by activating T help-er cells [35]. However, it was reported that the expression of IL-12 in U9378 macrophages was not increased by P. gin-givalis infection [36], and in the current study, *P. gingivalis* infection did not induce a substantial IL-12 increase in young BMDMs, but caused an obvious increase in old BMDMs, which indicated that infection of old cells might prompt the induction of adaptive immunity. These results suggested that aging induces the overexpression of infection-related cytokines and that these overexpressions may be markers of immunosenescence.

IL-1β plays an important role in the pathology of periodontitis, and thus, we examined the effect of aging on the signal pathway responsible for IL-1β production in the presence of *P. gingivalis*. Levels of NLRP3, AIM2, ASC, and pro-caspase1 components of the NLRP3/AIM2 complex were higher in *P. gingivalis*-infected old BMDMs and THP-1 macrophages than in their young cells (Figure 4A,B). Furthermore, ASC specks were higher in *P. gingivalis*-infected old THP-1 macrophages (Figure 4C,D). In particular, the NLPR3 inflammasome is associated with aging and age-related diseases, including Alzheimer’s disease [37], and metabolic disease [38]. In present study, we also showed that periodontal inflammation in the aging process is also implicated with the NLRP3 inflammasome complex. NLRP3 can be considered as a therapeutic target for inflammaging and age-related diseases.

Finally, we tested the effects of aging in a *P. gingivalis*-infected mouse model. We found that basal bone loss in non-infected old mice was greater than in non-infected young mice. Furthermore, crown cusp abra-sion and alveolar bone loss were greater in infected old mice than in infected young mice (Figure 5). In a clinical report, loss of alveolar bone in healthy individuals started at age 35 and increased gradually to age 85 [39]. Another study reported that osteoclast activity was greater in old (50-week-old) mice than in young (5-week-old) mice [40]. Our results indicate that *P. gingivalis* infection has a greater effect on alveolar bone resorption in old mice.

In summary, inflammatory cytokine production and inflammasome activation were elevated in *P. gingivalis*-infected senescent macrophage cells. Moreover, *P. gingivalis* infection accelerates bone resorption in old mice than young mice. Furthermore, our findings show that inflammatory reactions progress more rapidly and are more severe in the elderly. This study improves understanding of the involvement of senescent cells in the pathogenesis of periodontitis in older adults and supports the suggestion that age-associated pathological changes contribute to periodontitis and might explain the increased prevalence of periodontitis in the elderly.

## Figures and Tables

**Table 1:** Clinical parameters of healthy subjects and young/old patients with periodontitis.

		Healthy (N= 55)	Periodontitis Young (N = 29)	Periodontitis Old (N = 30)	df	χ^2^	*p*-Value *
PD	Mean	2.43	4.09	3.10	2	55.841	7.448 × 10^13^
	S.D.	0.17	1.27	0.98			
CAL	Mean	2.43	4.46	3.65	2	83.163	<2.2 × 10^16^
	S.D.	0.12	1.46	0.98			
GI	Mean	0.12	1.12	0.82	2	76.06	<2.2 × 10^16^
	S.D.	0.12	0.58	0.45			
PI	Mean	17.09	63.14	60.32	2	67.582	2.112 × 10^15^
	S.D.	14.69	27.97	20.26			

PD: probing depth; CAL: clinical attachment level; GI: gingival index; PI: plaque index; S.D.: standard deviation; *: Kruskal–Wallis test.

## Data Availability

Data for this study, though not available in a public repository, will be made available upon reasonable request.

## Abbreviations

BMDMs	Bone-marrow-derived cells
IL-1β	Interleukin-1β
NLRP3	NLR Family Pyrin Domain-Containing 3
AIM2	Absent in melanoma 2
PD	Probing depth
CAL	Clinical attachment level
GI	Gingival index
PI	Plaque index
MOI	Multiplicity of infection
